# Practice pattern variation: treatment of pelvic organ prolapse in The Netherlands

**DOI:** 10.1007/s00192-021-04968-8

**Published:** 2021-09-06

**Authors:** Rosa A. Enklaar, Mèlanie N. van IJsselmuiden, Joanna IntHout, Stefan J. H. Haan, Olivier G. A. M. Rijssenbeek, Rolf H. Bremmer, Hugo W. F. van Eijndhoven

**Affiliations:** 1 Radboud Institute for Health Sciences, Department of Obstetrics and Gynecology, Radboud University Medical Center, Geert Groote plein Zuid 10, 6525 GA Nijmegen, The Netherlands; 2 Department of Obstetrics and Gynecology, Zuyderland Medical Center, Heerlen, The Netherlands; 3 Department of Obstetrics and Gynecology, Isala, Zwolle, The Netherlands; 4 Radboud Institute for Health Sciences, Radboud University Medical Center, Nijmegen, The Netherlands; 5 LOGEX, Amsterdam, The Netherlands

**Keywords:** Pelvic organ prolapse, Uterine preservation, Hysterectomy, Practice pattern variation

## Abstract

**Introduction and hypothesis:**

Great variety in clinical management of pelvic organ prolapse (POP) has been described over the last years. Practice pattern variation (PPV) reflects differences in care that cannot be explained by the underlying condition. We aim to explore whether PPV in management of POP in The Netherlands has changed between 2011 and 2017.

**Methods:**

We conducted a multicenter cohort study, using prospective routinely collected benchmark data from LOGEX, a healthcare analytics company (Amsterdam, The Netherlands). Data of patients with a diagnosis POP from 50 hospitals (16 teaching and 34 non-teaching hospitals) were collected for the years 2011 and 2017. All treatments were categorized into three groups: conservative treatment, uterus-preserving or uterus-removing surgery. Using meta-analysis, we evaluated whether the proportions of conducted treatments changed over time and estimated the between-center variation (Cochran’s Q), reflecting the PPV in 2011 and 2017. This variation was analyzed using F-tests.

**Results:**

Compared to 2011, referral for POP in 2017 decreased by 16.2% (−4505 patients), and the percentage of hysterectomies decreased by 33.6% (*p* < 0.0001). The PPV of POP surgery decreased significantly by 47.2% (*p* = 0.0137) and of hysterectomies by 41.5% (*p* = 0.0316).

**Conclusions:**

We found a decline in PPV for POP surgery between 2011 and 2017. Furthermore, the number of surgical interventions decreased, which was mostly due to a decline of hysterectomies. This indicates a shift toward more conservative therapy and uterus preservation. A further reduction of PPV would be beneficial for the quality of health care.

## Introduction

The lifetime risk of undergoing surgery for pelvic organ prolapse (POP) or urinary incontinence (UI) is 20% by the age of 80 [1]. Because of the aging population and the increase of obesity rates, an expected increase of patients with POP seeking treatment in the near future is likely [2]. According to the NICE guideline, the first choice of treatment for POP is conservative, e.g., a pessary or physiotherapy [3]. Surgery is offered to women whose symptoms do not improve with conservative treatment or who prefer surgical therapy [3]. A wide range of surgical interventions is available. Until today, the procedure of first choice for uterovaginal prolapse in The Netherlands is a vaginal hysterectomy (VH) with vaginal vault suspension [4], followed by uterus-preserving techniques [vaginal sacrospinous hysteropexy (SSH) or Manchester Fothergill repair (MF)]. Apart from symptoms, type and severity of the prolapse, the experience and preference of the gynecologist and the patient’s wishes influence the choice of intervention [5].

The availability of conservative therapy and different surgical options can lead to variation in treatment between physicians and hospitals. This phenomenon is called practice pattern variation (PPV). PPV is defined as the difference in care that cannot be explained by the underlying medical condition. It can be caused by lack of evidence-based standards, non-compliance toward these standards or a difference in skills and resources. PPV can lead to under- and overtreatment and therefore could introduce unnecessary risks of medication or surgery, or patients might not receive adequate treatment for their medical condition [6]. In general, PPV is associated with higher costs. The first report of regional variation in surgical practice dates from 1938, describing the incidence of tonsillectomy in school children [7]. This study showed differences in local surgical rates varying between 4% and 45% in a small region. Today, there are many examples of PPV in different areas of healthcare. Recently, we described PPV in the treatment of POP in The Netherlands [8]. Although The Netherlands is a small country with a limited number of (uro)gynecologists and hospitals, the degree of PPV was impressive and higher than expected. Besides the choice between operative and conservative therapy, PPV was noticed in the type of surgery and operative techniques. Especially the choice between hysterectomy and uterus-preserving POP surgery showed a wide variety. This variation could not be explained by differences in patient population and characteristics or differences in skills or resources. Whether or not lack of evidence for the optimal treatment or lack of evidence-based guidelines plays a role in this PPV is unclear.

In recent years, many studies have been published concerning uterus-preserving surgery in case of POP and these techniques have become more popular. Long-term follow-up also showed that SSH possibly has a better composite outcome of success compared to hysterectomy [9, 10]. A Danish register-based study showed a decrease of vaginal hysterectomies and an increase of uterus-preserving techniques such as SSH and MF [11]. They also claimed MF to be superior to VH for treating POP [12]. Furthermore, when equal outcome is expected, women are more likely to choose a uterus-preserving technique over vaginal hysterectomy [13, 14]. These new insights into uterus-preserving surgical techniques might have an impact on the current surgical approach to uterine descent. The aim of this study is to explore whether PPV in management of POP in The Netherlands has changed in recent years.

## Materials and methods

A multicenter cohort study was performed using prospective routinely collected data retrieved from the ‘Benchmark Database’ serviced by LOGEX, a Dutch healthcare data analytics company. The data contain patient-level information on diagnosis, care activities and discharges, complemented by several patient characteristics. These data are primarily generated and used for reimbursement purposes and are also considered an accurate source for research into the quality and costs of healthcare [15, 16]. The benchmark database used for this study includes 50 out of 74 Dutch hospitals. Health care insurance is mandatory in The Netherlands, and all invoice data given to health insurance companies are based on health care declaration codes. Each medical condition has a specific declaration code developed by the Dutch Healthcare Authority (NZA); the official nationwide healthcare code for POP and UI is G25 [17]. Within this healthcare declaration code conservative treatment and surgical procedures for both POP and UI performed by gynecologists are registered. The number of surgical interventions for UI has been stable over the last years [17] because of clear guidelines. Therefore, the focus of this study is on the potential change of PPV for POP in The Netherlands.

Data were collected for all patients with a G25 diagnosis from the participating hospitals for the years 2011 and 2017. The latter is the most recent year with a complete data set available for analysis. The specific care activity codes that were included in the analysis are shown in Appendix Table 2. All operations listed were registered only for POP complaints and not for other benign symptoms. These included vaginal surgery with and without mesh and laparoscopic POP surgery. Once a patient is referred to the gynecologist for POP, this patient and the corresponding care activity codes are included in the year of the first G25 occurrence (2011 or 2017). Therefore, a patient can only be included once, even if the treatment continues for > 1 year. The health care activities of each patient were collected until 2.5 years after inclusion for both 2011 and 2017. The total number of patients included in 1 year corresponds with the incidence rate in that year.

The treatment of a patient was assigned to one of three categories—conservative treatment, uterus-preserving surgery and uterus-removing surgery—based on the presence or absence of care activities. The occurrence of the care activity had to be linked to a G25 episode. Patients could have multiple (surgical) treatments (e.g., first uterus-preserving surgery and subsequently uterus-removing surgery). In those cases, allocation was determined by the treatment with the highest impact. The impact of the treatment was considered low in case of conservative treatment, intermediate with uterus-preserving treatment and high for hysterectomy. Hence, a hysterectomy was assumed to have and categorized as having the highest impact.

For the analysis, hospitals were categorized as teaching hospitals, including university hospitals and non-teaching hospitals. For each hospital, the pelvic floor surgical rate was calculated and defined as the percentage of all women referred to that hospital for POP complaints who underwent a surgical procedure (Table 1). Furthermore, the number of hysterectomies was calculated as a percentage of all surgical procedures for uterine descent. The surgery rate was also differentiated for teaching and non-teaching hospitals.

**Table 1:** Overall pelvic organ prolapse (POP) surgery and hysterectomies in 2011 versus 2017

		2011	2017	Relative difference (95% CI)
All hospitals (*n* = 50)	Number of patients in G25^a^	27,727	23,222	−16.2%
	POP surgery^b^	9500 (34.3%)	6390 (27.5%)	−17.2% (−21.4 to −12.8)
	Median (range^c^) of POP surgeries per hospital	180 (32–479)	113 (18–372)	
	Hysterectomy^d^	2560 (26.8%)	1213 (19.0%)	−33.6% (−38.9 to −27.7)
	Median (range) of hysterectomies per hospital	43 (3–141)	17 (2–87)	
Teaching (*n* = 16)	Number of patients in G25	12,636	10,626	−15.9%
	POP surgery	4168 (33.0%)	2773 (26.1%)	−14.2% (−20.3 to −7.7)
	Hysterectomy	882 (21.2%)	396 (14.3%)	−28.4% (−34.5 to −21.8)
Non-teaching (*n* = 34)	Number of patients in G25	15,091	12,596	−16.5%
	POP surgery	5332 (35.3%)	3617 (28.7%)	−22.3% (−26.4 to −18.0)
	Hysterectomy	1678 (31.3%)	817 (22.6%)	−43.7% (−52.9 to −32.7)

^a^*Number of patients in G25: number of patients who registered for the first time with POP complaints*

^b^*POP surgery and hysterectomies: number of operations in absolute numbers and percentages. Percentage of POP surgery represents the number of surgical therapies within the G25 care product*

^c^*Range: minimum–maximum*

^d^*Percentage of hysterectomies is presented as percentage of total number of POP surgeries*

*P* values for differences between 2011 and 2017: *p* < 0.0001 for POP and/or UI surgery; *p* < 0.0001 for hysterectomy

*P* value for subgroup differences between teaching and non-teaching hospitals: *p*  = 0.225 for POP surgery; *p* = 0.177 for hysterectomy

We estimated the relative risk of POP surgery and from this the relative difference in the number of POP surgeries in 2017 compared to 2011. A random-effects meta-analysis was applied, using the Mantel-Haenszel method with the Hartung-Knapp-Sidik-Jonkman approach[18], taking the type of hospital (teaching or non-teaching) into account. Standard errors were corrected for the fact that the random sample of 50 hospitals was taken from a total of 74 hospitals by multiplying the variance with a factor (74–50)/(74–1) = 0.329. Confidence intervals and corresponding *p* values were based on the corrected standard errors. Differences between the teaching and non-teaching hospitals were estimated with a chi-squared test based on Cochran’s Q. A similar analysis was executed for the number of hysterectomies.

To estimate the changes in PPV, two separate random-effects meta-analyses were conducted to estimate the pooled percentage of POP surgeries and the variability for 2011 and 2017 respectively. In these models, the variation (heterogeneity) between hospitals was estimated with a REML estimator for τ^2^ and quantified with a 95% prediction interval that predicts with 95% certainty the expected proportion of operations in a randomly selected hospital similar to the hospitals in the study. As we aimed for an estimate of τ on the proportion scale, we did not transform the proportions in the meta-analysis. Therefore, the τ can be interpreted as a standard deviation (SD) of the proportions between hospitals. However, τ differs from an SD as the τ is corrected for imprecision in the proportions of surgeries because of the limited size of the hospitals, whereas an SD would directly use the point estimates of the proportions of the hospitals.

The larger the τ and the wider the resulting prediction interval, the larger the PPV is [19]. To evaluate whether there was a decrease in PPV, we estimated Cochran’s Q, the weighted sum of squared differences between the proportions of the individual hospitals and the pooled proportion across hospitals, with the weights being those used in the random-effects model. To test the significance of the change in PPV, an F-test with 49 and 49 degrees of freedom was applied on the ratio of the two Q’s. Two-sided *p* values < 0.05 were considered statistically significant. The analysis was performed with the R statistical software (version 3.6.2), using the meta package version 4.11.0 [20].

## Results

In total, data of 50 hospitals were available: 16 teaching hospitals (including 2 university hospitals) and 34 non-teaching hospitals. Compared to 2011, referral for POP decreased in 2017 by 16.2%. Furthermore, a decrease in operations was noticed in each hospital and overall. The percentage of hysterectomies decreased with 33.6% [95% confidence interval (CI) –38.9 to −27.7, *p* < 0.0001]. This decrease was almost twice as large as the decrease in pelvic floor surgeries (−17.2%, 95% CI −21.4 to −12.8, *p* < 0.0001). There was no significant difference between the teaching and non-teaching hospitals (Table 1).

### Practice pattern variation (PPV)

**Fig. 1:**
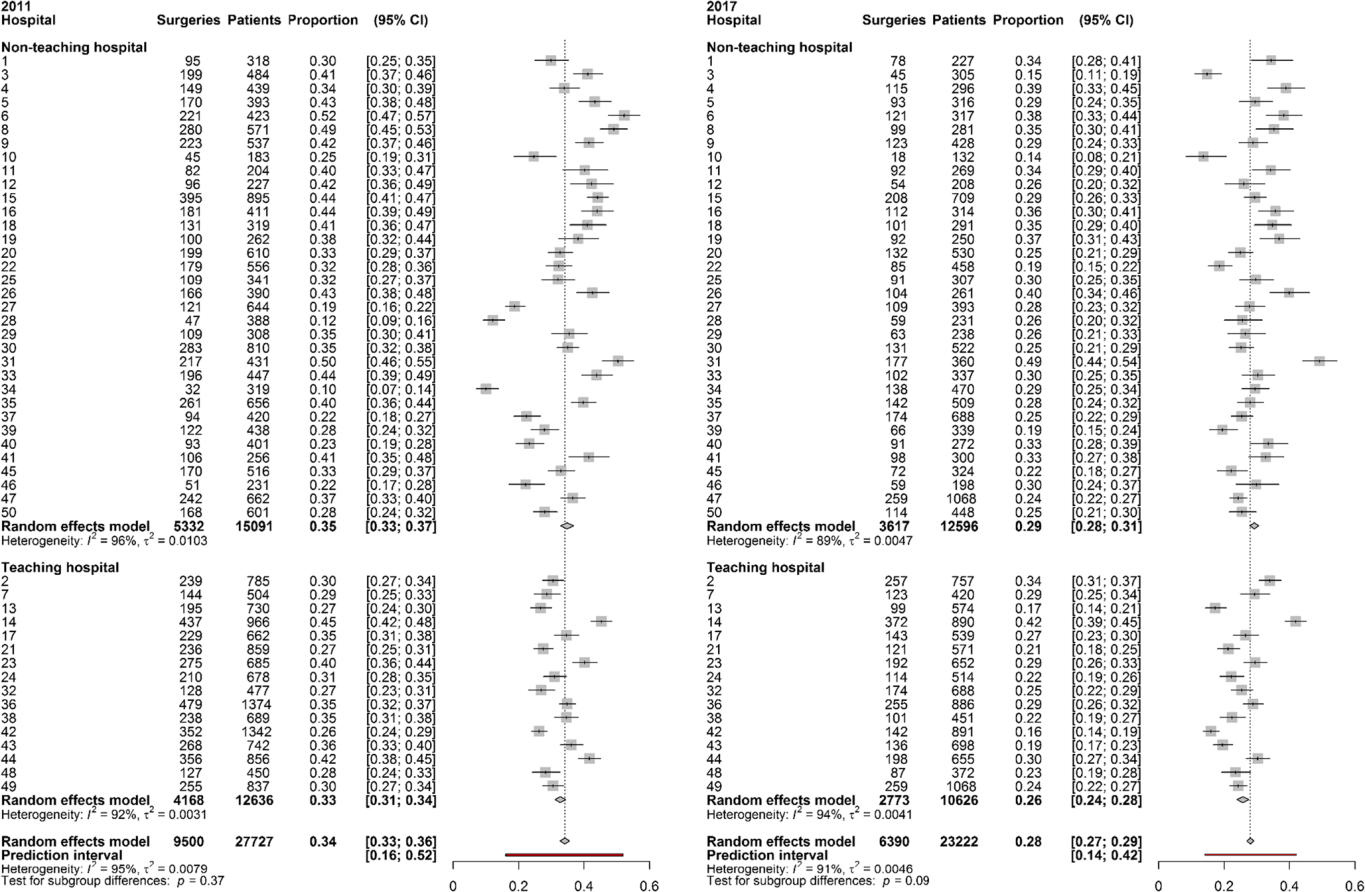
Forest plots of the number of surgeries for POP per hospital (16 teaching hospitals, 34 non-teaching hospitals) for 2011 and 2017

Figure 1 shows the proportion of patients who had surgeries for POP in 2011 and 2017 for each hospital (teaching and non-teaching). The 95% prediction intervals (PI) show the wide range of surgery rates that can be expected in a randomly selected hospital (red line in Fig. 1). When comparing the length of the PI of 2011 (95% PI 16.0–52.1%) with 2017 (95% PI 14.2–41.8%), a decrease in PPV for pelvic floor surgery between the 2 years is found. Also, the τ decreased from 8.9% (95% CI 7.3–11.2%) in 2011 to 6.8% (95% CI 5.6–8.7%) in 2017. τ can be interpreted as the standard deviation (SD) of the percentages between the hospitals. Comparing the PPV of both years using a test of the variances based on Cochran’s Q shows a significant decline of 47.2% (*p* = 0.0137). This is mainly related to a decline in PPV in the non-teaching hospitals.

**Fig. 2:**
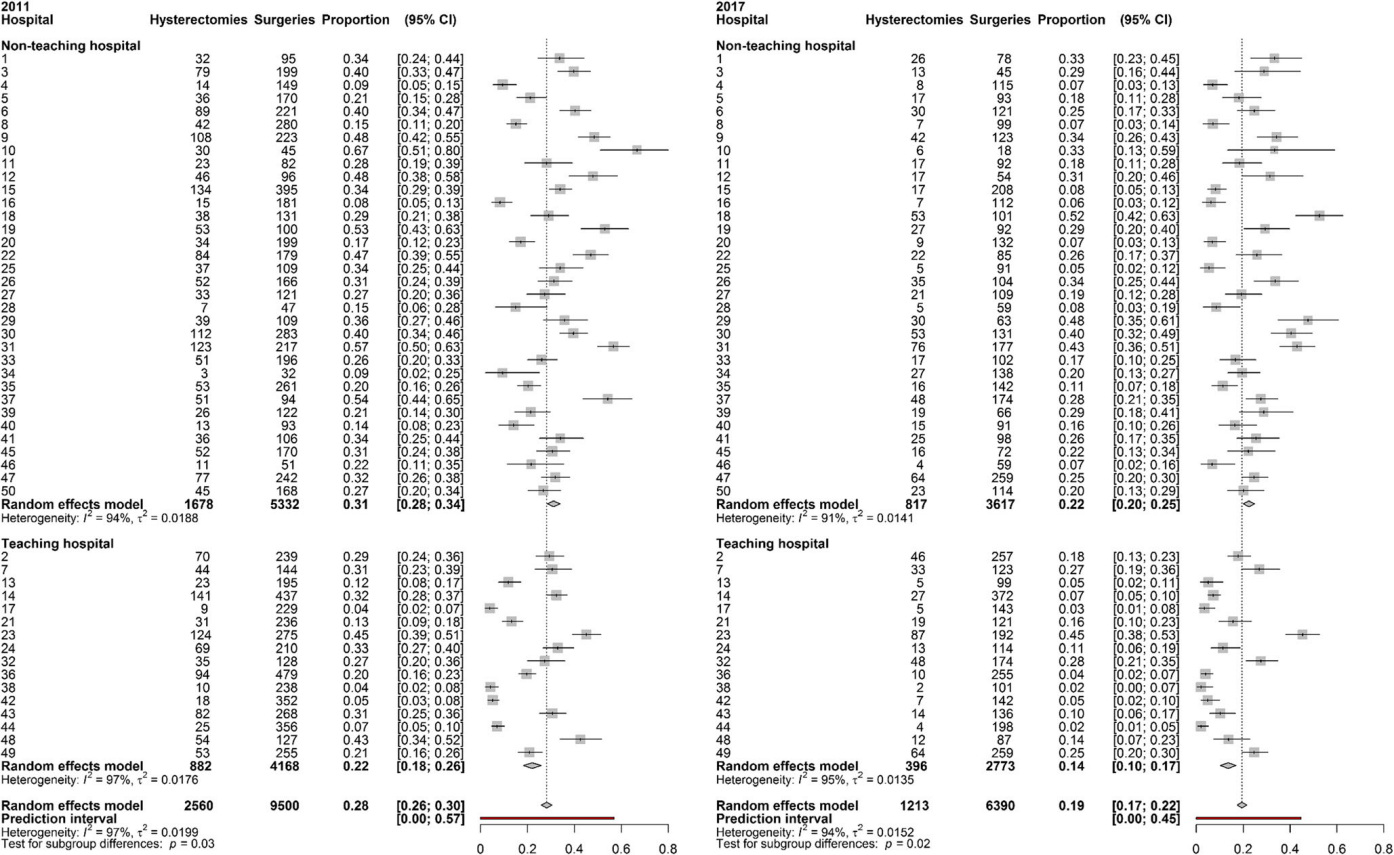
Forest plots of the number of hysterectomies for POP per hospital (16 teaching hospitals, 34 non-teaching hospitals) for 2011 and 2017

In Fig. 2, the number of hysterectomies in 2011 and 2017 is displayed for each hospital. The 95% PI for hysterectomies in 2011 and 2017 became shorter (2011: 95% PI 0.0–56.9%, 2017: 95% PI 0.0–44.6; red line). This is also reflected by Cochran’s Q, which shows a decrease of 41.5% (*p* = 0.0316). Furthermore, the τ decreased from 14.1% (95% CI 11.7–17.9%) in 2011 to 12.3% (95% CI 10.1–15.7%) in 2017.

## Discussion

In this study we describe changes in PPV for POP surgery in The Netherlands for 2011 to 2017. To our knowledge, this is the first time that changes in PPV were analyzed. We found a significant decline in PPV in operative treatment for POP between the reviewed hospitals, especially in the non-teaching hospitals. That is to say, the balance between conservative and operative treatment for POP showed less variation nationwide. Moreover, PPV in vaginal hysterectomy for POP decreased in favor of uterus-preserving surgery. Because the number of operations for UI were stable over the years [17], we believe that the changes in POP treatment are accountable for the changes in PPV.

The decrease of the surgical interventions for POP might indicate that more patients have chosen for conservative therapy. The decrease of hysterectomies for POP might imply a shift toward uterus-preserving surgical procedures. This assumption also seems to be consistent with the recent developments and publications in favor of uterus-preserving surgery for POP [9, 11].

One of the main reasons for PPV in developed countries is the absence of clearly defined guidelines, non-compliance with guidelines or lack of evidence for the optimal treatment. A clear guideline on the treatment of UI has already been available in The Netherlands for more than a decade. However, it took until 2014 for the first guideline for management of POP to be published by the Dutch Society for Obstetrics and Gynecology (NVOG). Conservative treatment is considered to be the first choice of treatment for POP. The guideline stated that there is not enough scientific evidence to show a difference in recurrence rate between uterus-preserving and uterus-removing surgery [21, 22]. However, since 2015 several studies have shown that SSH and MF are at least non-inferior and after 5-year follow-up possibly have some benefits compared to VH [9, 11, 12, 23]. It is plausible that the guideline and growing scientific evidence for uterus-preserving techniques have contributed to the reduction of PPV in POP treatment as we have described.

Multiple studies have shown a growing preference of women for uterus preservation [13, 14]. Patients considered restoration of ‘normality,’ symptom relief and physical improvement to be the most important factors [24]. However, physician’s preference has a major impact on the counseling of the patient and therefore on the choice of treatment. Surgeons tend to pick data and use studies that confirm their own beliefs, which causes confirmation bias. These beliefs are often based on their own experiences or on those of their colleagues [25]. Counseling might also be influenced by the capacities of the surgeon. Ideally, every gynecologist should be able to offer at least the most common surgical techniques for POP. In reality, patients will only be counseled for the techniques the gynecologist is most familiar and experienced with [25]. Fortunately, patient preference and shared decision-making are gaining more attention in the medical field. Optimizing the shared decision-making process and minimizing the difference in surgical approaches should contribute to a further decrease of PPV. To provide non-ambiguous counseling and care, a future perspective might be in centralized pelvic floor centers.

Recent publications on uterus-preserving operative techniques have provided more insight into these treatment modalities. In a Danish register-based study, a decrease of vaginal hysterectomies and an increase of uterus-preserving techniques such as SSH and MF were described [11]. A matched cohort extracted from the same database (DugaBase) showed that the risk of recurrence in any compartment was higher after VH (18.3%) compared to MF (7.8%). The authors suggested that if there is no other indication for hysterectomy, VH should never be the first choice for surgical treatment of POP [12]. In the coming years, the results of an ongoing randomized controlled trial concerning two uterus-preserving surgical techniques for uterine descent (SSH and MF) will be published [26]. This will provide new scientific evidence on the role of these operative techniques that can contribute to further reduction of PPV. Despite several publications concerning PPV in urogynecology, not one has described a change in PPV based on a nationwide registration. The known studies are based on surveys among gynecologists and mainly describe operative techniques and interoperative differences [27, 28]. Some other studies have demonstrated PPV in the treatment of vaginal vault prolapse or determined compliance to evidence-based practices in the (surgical) management of POP [29, 30].

This study has some strengths and limitations. In The Netherlands the registration of claims data through care products (a combination of care activities) is mandatory, and Dutch physicians are financially depending on registering the correct activities. Therefore, it can be assumed that the data will be complete and reliable. Also, the follow-up period of 2.5 years after the first registration per patient is of adequate length. Out of all patients who have to undergo an operation for POP, 87% will be operated on within the 1st year and 97% will be operated on within 2 years.

A limitation of this study is that we were not able to separate first time surgery from recurrent surgery and, as a consequence, could not differentiate between primary and recurrent surgery. In both 2011 and 2017 all patients included in our database were registered as new patients. In The Netherlands, a patient must be referred again by the general practitioner when 1 year has passed without treatment by the gynecologist. Therefore, it was not known whether patients had undergone previous hysterectomy or operations for POP in the past.

In conclusion, in The Netherlands the number of patients referred for POP has decreased over the last years. Both conservative treatment and uterus-preserving surgery increased during the same period. In line with these trends, there was a significant reduction of PPV in both pelvic floor surgery and VH for POP. It is likely that new insights in surgical management of POP and the introduction of an evidence-based guideline have contributed to this reduction. Patient-centered care, the use of structured decision aids and shared decision-making are growing trends and might play an important role in further reduction of PPV in the future.

## Appendix

**Table 2.** Care activity codes included in the analysis

**Pelvic organ prolapse surgery**	
37263	General prolapse surgery, anterior and/or posterior colporrhaphy. With or without plication of the vaginal fascia for minor UI
37264	Prolapse surgery, anterior and/or posterior colporrhaphy, and portio-amputation
37268	Vaginal reconstructive prolapse surgery: anterior or posterior colporrhaphy, with single mesh (TVM-procedure), or intra-vaginal sling (IVS)
37370	Enterocele repair, abdominal or vaginal
37380	Abdominal retroperitoneal fixation of the vaginal vault (or procedure according to Rust)
37383	Laparoscopic sacropexy, including anterior and/or posterior colporrhaphy
37384	Laparoscopic sacropexy, excluding anterior and/or posterior colporrhaphy
37385	Vaginal sacrospinous hysteropexy, including anterior and/or posterior colporrhaphy
37386	Vaginal sacrospinous hysteropexy, excluding anterior and/or posterior colporrhaphy
35021	Abdominal sacropexy for rectal prolapse
37131 *	Vaginal hysterectomy
37113 *	Laparoscopic hysterectomy, LAVH/LASH
37265 *	Vaginal hysterectomy, including anterior and/or posterior colporrhaphy

*Care activity codes used to differentiate identify the hysterectomy group
